# Molecular identification of new *Trypanosoma evansi* type non-A/B isolates from buffaloes and cattle in Indonesia

**DOI:** 10.1590/S1984-29612024033

**Published:** 2024-06-28

**Authors:** Didik Tulus Subekti, Lucia Tri Suwanti, Dyah Ayu Kurniawati, Mufasirin Mufasirin, Sunarno Sunarno

**Affiliations:** 1 Veterinary Science Doctoral Program, Faculty of Veterinary Medicine, Airlangga University, Kampus C Mulyorejo, Surabaya, East Java, Indonesia; 2 Center for Biomedical Research, Research Organization for Health, National Research and Innovation Agency, Cibinong Science Center, Bogor West Jawa Province, Indonesia; 3 Division of Parasitology, Faculty of Veterinary Medicine, Airlangga University, Kampus C Mulyorejo, Surabaya, East Java, Indonesia; 4 Center for Veterinary Instrument Standard Testing – CVIST, Agency for Standardization of Agricultural Instruments, Indonesian Ministry of Agriculture, Bogor, West Jawa Province, Indonesia

**Keywords:** Trypanosoma evansi, genotype, type non A/B, algorithm, molecular identification, Trypanosoma evansi, genótipo, tipo não A/B, algoritmo, identificação molecular

## Abstract

*Trypanosoma evansi* is reportedly divided into two genotypes: types A and B. The type B is uncommon and reportedly limited to Africa: Kenya Sudan, and Ethiopia. In contrast, type A has been widely reported in Africa, South America, and Asia. However, *Trypanosoma evansi* type non-A/B has never been reported. Therefore, this study aims to determine the species and genotype of the *Trypanozoon* subgenus using a robust identification algorithm. Forty-three trypanosoma isolates from Indonesia were identified as *Trypanosoma evansi* using a molecular identification algorithm. Further identification showed that 39 isolates were type A and 4 isolates were possibly non-A/B types. The PML, AMN-SB1, and STENT3 isolates were likely non-A/B type *Trypanosoma evansi* isolated from buffalo, while the PDE isolates were isolated from cattle. Cladistic analysis revealed that Indonesian *Trypanosoma evansi* was divided into seven clusters based on the *gRNA-kDNA* minicircle gene. Clusters 6 and 7 are each divided into two sub-clusters. The areas with the highest genetic diversity are the provinces of Banten, Central Java (included Yogyakarta), and East Nusa Tenggara. The Central Java (including Yogyakarta) and East Nusa Tenggara provinces, each have four sub-clusters, while Banten has three.

## Resumo

*Trypanosoma evansi* é reportado como dividido em dois genótipos: tipos A e B. O tipo B é incomum e reportado como limitado à África: Quênia, Sudão e Etiópia. Em contraste, o tipo A tem sido amplamente relatado na África, América do Sul e Ásia. No entanto, *Trypanosoma evansi* tipo não-A/B nunca foi relatado. Portanto, este estudo tem como objetivo determinar a espécie e o genótipo do subgênero *Trypanozoon*, utilizando-se um algoritmo robusto de identificação. Quarenta e três isolados de tripanosoma da Indonésia foram identificados como *Trypanosoma evansi*, usando-se um algoritmo de identificação molecular. A identificação adicional mostrou que 39 isolados eram do tipo A e 4 isolados eram, possivelmente, do tipo não A/B. Os isolados PML, AMN-SB1 e STENT3 foram, provavelmente, *Trypanosoma evansi* do tipo não A/B isolado de búfalos, enquanto os isolados de PDE foram isolados de bovinos. A análise cladística revelou que o *Trypanosoma evansi* indonésio foi dividido em sete grupos baseados no gene do minicírculo gRNA-kDNA. Os clusters 6 e 7 foram divididos cada um em dois subclusters. As áreas com maior diversidade genética são as províncias de Banten, Java Central (incluindo Yogyakarta) e East Nusa Tenggara. As de Java Central (incluindo Yogyakarta) e East Nusa Tenggara têm, cada uma, quatro subgrupos, enquanto Banten tem três.

## Introduction

Species identification in the *Trypanozoon* subgenus based on morphology and molecular markers still causes disputes among researchers. It is difficult to morphologically identify the three *Trypanozoon* subgenus species because of their morphological similarities (Li et al., 2006; Sánchez et al., 2016; Wen et al., 2016; Gizaw et al., 2017). Their molecular identification is similarly challenging, with some commonly used primer pairs such as ITS1 and ITS2 or TBR known to detect pan-trypanosomes (WOAH, 2021). The three primer pairs can detect a broad range of species, such as *Trypanosoma congolense*, *Trypanosoma simiae*, *Trypanosoma vivax*, *Trypanosoma theileri*, *Trypanozoon* subgenus, and several other species (Salim et al., 2014; Isaac et al., 2016; Alanazi et al., 2018; Gaithuma et al., 2019; Marsela et al., 2020). Therefore, it is more suitable to screen based on their DNA sequence.

However, the ESAG6/7 or RoTat1.2 primer pairs are known to only identify up to the *Trypanozoon* subgenus (WOAH, 2021). The ESAG6/7 primer pair amplifies the expression site associated gene 6/7 (ESAG6/7) that encodes the transferrin receptor protein (Tf-R), which comprises ESAG6 and ESAG7 protein subunits (Gerrits et al., 2002; Kariuki et al., 2019). Primer pairs targeting variant surface glycoprotein (VSG) Rode *Trypanozoon* antigen type (RoTat) 1.2 have also been reported to identify only the *Trypanozoon* subgenus (El-Naga et al., 2012; WOAH, 2021). VSG is a structural layer of glycoprotein that coats the entire cell surface of *Trypanosoma* sp. (Sudan et al., 2017; Gaur et al., 2021). RoTat 1.2 is VSG’s predominant variant antigen type (Gaur et al., 2021). Several RoTat 1.2 primer pairs have different nucleotide sequences and have been used for different purposes, including identifying *Trypanosoma evansi* genotypes (Birhanu et al., 2016).

However, some primer pairs can be used to distinguish *Trypanosoma brucei* from *T. evansi* by targeting the kinetoplast DNA (kDNA) minicircle gene (Artama et al., 1992). *T. evansi* can also be distinguished from *T. brucei* and *Trypanosoma equiperdum* by targeting the kDNA maxicircle gene (Li et al., 2007). It has recently been reported to use several primer pairs successively for species identification (Subekti et al., 2023). Therefore, the appropriate algorithm design will greatly increase the accuracy of molecular identification of *Trypanozoon* species. Genetically, *T. evansi* has also been reported to be divided into two genotypes: types A and B (Birhanu et al., 2016; Boushaki et al., 2019; Li et al., 2020). Molecular identification for genotype classification relies on two primer pairs: ILO7957/8091 targeting the VSG RoTat 1.2 gene and EVAB targeting the kDNA minicircle type B (Njiru et al., 2006; Birhanu et al., 2016; Boushaki et al., 2019).

*T. evansi* type B is uncommon and reportedly limited to Africa: Kenya, Sudan, Chad, and Ethiopia (Birhanu et al., 2016; Boushaki et al., 2019). In contrast, *T. evansi* type A has been widely reported in Africa, South America, and Asia (Njiru et al., 2006; Birhanu et al., 2016). This study aims to identify *T. evansi* from Indonesian isolates with molecular identification algorithms while establishing genotypes and their genetic diversity.

## Materials and Methods

### Trypanosome and DNA extraction

Forty-three trypanosome isolates from several regions of Indonesia were grown in Deutschland, Denken, and Yoken (DDY) mice. When their parasitemia was high, the mice were euthanized, and blood was collected by heart puncture. Next, *Trypanosoma*-containing blood was purified using the Toyopearl 650M DEAE-methacrylate polymer (Tosoh Bioscience, Philadelphia, PA, USA; Subekti et al., 2023). DNA was extracted from pure trypanosomes using DNAzol (Molecular Research Center Inc., Cincinnati, OH, USA) according to the manufacturer’s instructions. All extracted DNA was stored in the freezer (−20°C) until needed.

### Species identification

**Figure 1:**
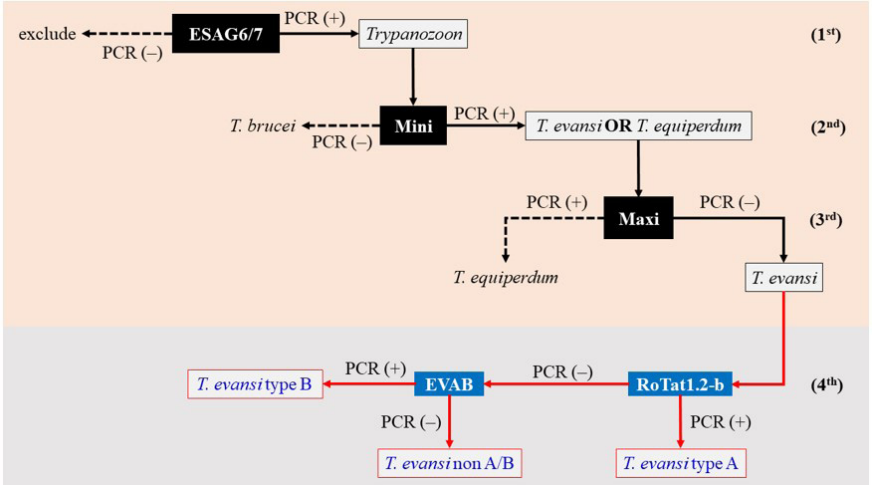
Molecular identification algorithm for *Trypanozoon* subgenus species. Mini = gRNA-kDNA minicircle; Maxi = ND5-kDNA maxicircle; RoTat 1.2-b = ILO7957/8091.

*T. evansi* species were identified using three primer pairs sequentially: ESAG6/7 (WOAH, 2021), guide RNA (gRNA)-kDNA minicircle (Artama et al., 1992), and ND5-kDNA maxicircle (Li et al., 2007). The molecular identification algorithm was performed according to the guidelines in Figure 1. After the *T. evansi* isolates were identified, their genotypes were determined using two primer pairs: ILO7957/8091 and EVAB (Birhanu et al., 2016; Boushaki et al., 2019).

### PCR primer and program

The primers used in the study and their amplification program are briefly described in Table 1. Polymerase chain reaction (PCR) was performed using a GTC96S, 96-well Thermal Cycler (Cleaver Scientific, Rugby, Warwickshire, UK). The 50 μL reaction mixture contained 1 µL (100 ng/µL) DNA, 1 µL (20 µM) of each primer (forward and reverse), 25 µL of MyTaq™ HS Red Mix 2x (Meridian Life Science Inc., Memphis, TN, USA), and 22 μL of nuclease-free water (Promega, Madison, WI, USA).

**Table 1:** The nucleotide sequences and PCR programs of the PCR primers used in this study.

Primer name	Nucleotide sequence (5’ to 3’)	Source	PCR program
ESAG6/7	F: ACATTCCAGCAGGAGTTGGAG	Holland et al. (2001)	1’ at 94^o^C, 35 cycles:
	R: CACGTGAATCCTCAATTTTGT		[1’ at 94^o^C, 2’ at 55^o^C, 2’ at 72^o^C], and 10’ at 72^o^C
MINI	F: CAACGACAAAGAGTCAGT	Artama et al. (1992)	1’ at 94^o^C, 35 cycles:
	R: ACGTGTTTTGTGTATGGT		[1’ at 94^o^C, 2’ at 55^o^C, 2’ at 72^o^C], and 10’ at 72 ^o^C
MAXI	F: TGGGTTTATATCAGGTTCATTTATG	Li et al. (2007)	1’ at 94^o^C, 35 cycles:
	R: CCTAATAATCTCATCCGCAGTACG		[1’ at 94^o^C, 1’ at 55^o^C, 2’ at 72^o^C], and 10’ at 72^o^C
RoTat 1.2-a	F: GCGGGGTGTTTAAAGCAATA	Claes et al. (2004);	4’ at 94^o^C, 35 cycles:
	R: ATTAGTGCTGCGTGTGTTCG	WOAH (2021)	[1’ at 94^o^C, 1’ at 59^o^C, 1’ at 72^o^C], and 5’ at 72^o^C
RoTat 1.2-b	F: GCCACCACGGCGAAAGAC	Urakawa et al. (2001);	1’ at 94^o^C, 35 cycles:
	R: TAATCAGTGTGGTGTGC	Birhanu et al. (2016)	[1’ at 94^o^C, 1’ at 52^o^C, 1’ at 72^o^C], and 5’ at 72^o^C
RoTat 1.2-c	F: CTGAAGAGGTTGGAAATGGAGAAG	Konnai et al. (2009);	1’ at 94^o^C, 35 cycles:
	R: GTTTCGGTGGTTCTGTTGTTGTTA	Salim et al. (2011)	[1’ at 94^o^C, 1’ at 59^o^C, 1’ at 72^o^C], and 5’ at 72^o^C
EVAB	F: CACAGTCCGAGAGATAGAG	Njiru et al. (2006)	5’ at 95^o^C, 30 cycles:
	R: CTGTACTCTACATCTACCTC		[1’ at 94^o^C, 1’ at 60^o^C, 1’ at 72^o^C], and 10’ at 72^o^C

**Notes:** MINI = gRNA-kDNA minicircle; MAXI = ND5-kDNA maxicircle; RoTat 1.2-b = ILO7957/8091; RoTat 1.2-c = TeRoTat920/1070.

The PCR product (amplicon) was electrophoresed in a 1.5% agarose gel with 1^st^ Base FloroSafe DNA stain (Axil Scientific Pte Ltd., Singapore) using the RunVIEW real-time gel visualization system (Cleaver Scientific) and visualized using a Clear View UV Transilluminator (Cleaver Scientific).

### Sequencing and cladogram construction

The PCR products were sequenced at Bioneer Corp. (Daejeon, Republic of Korea). The obtained nucleotide sequences were assessed for similarity to other trypanosome isolates using the Basic Local Alignment Search Tool (BLAST) from the US National Center for Biotechnology Information (Altschul et al., 1997). The possible identity of the trypanosome isolates was determined based on all nucleotide sequences in the BLAST alignments with the highest percentage sequence similarity and query coverage for each identified species.

The cladogram was constructed using two approaches. The first used the nucleotide sequence of the *gRNA-kDNA* minicircle gene, while the other used the binary data derived from the PCR results with primer pairs ESAG6/7, gRNA-kDNA minicircle (MINI), RoTat 1.2, and EVAB. The cladogram based on the nucleotide sequence of the *gRNA-kDNA* minicircle was constructed using CLC Sequence Viewer 8.0 (Qiagen, Copenhagen, Denmark) with the Neighbor-joining method using Jukes-Cantor nucleotide distance measurement and bootstrap analysis with 1000 replicates. The cladogram was visualized using The Interactive Tree Of Life (https://itol.embl.de) (Letunic & Bork, 2021). The cladograms based on the binary data were constructed using hierarchical cluster analysis (HCA) with Minitab (Minitab LLC, State College, PA, USA) with the average linkage method (Stevens & Godfrey, 1992).

## Results and Discussion

### Molecular identification and genotyping

Forty-three trypanosome isolates were identified as *T. evansi* using the molecular identification algorithm. The alignment of the nucleotide sequences of PCR amplicons with the ESAG6/7 primer pair showed sequence similarity to three species in the *Trypanozoon* subgenus. Sequence similarity to *T. evansi* ranged from 89.90% to 98.11%, *T. brucei* ranged from 91.33% to 98.33%, and *T. equiperdum* ranged from 84.69% to 97.17% (Table 2). These results are consistent with several reports that concluded that the ESAG6/7 primer pair could identify the *Trypanozoon* subgenus but not the species (Holland et al., 2001; Isobe et al., 2003; WOAH, 2021).

**Table 2:** Sequence similarity of Indonesian trypanosome isolates based on expression site-associated genes region 6 (ESAG6) and *gRNA-kDNA* minicircle genes.

No	Code	Host	Year	ESAG6 Sequence Similarity ( accession number)^φ^			*gRNA-kDNA* minicircle Sequence Similarity (accession number)φ,*	
				*T. evansi*	*T. brucei*	*T. equiperdum*	*T. evansi*	*T. equiperdum*
1	AMN-SB1	Buffalo	2013	97.50% (JF8942421)	98.33% (L07805)	94.17% (EU726386)	93.85% (M57462)	93.02% (EU155058)
2	KPG	Buffalo	1985	96.57% (KR858299)	97% (FM162581)	93.99% (EU726386)	96.19% (M81594)	96.12% (EU155058)
3	PML	Buffalo	1996	95.85% (JF894242)	96.68% (KC257414)	92.53% (EU726386)	95.05% (M57462)	94.15% (EU155058)
4	STENT1	Buffalo	2012	94.71% (KR858301)	96.09% (EU726442)	91.96% (EU726386)	95.31% (M81594)	92.06% (M14763)
5	STENT5	Buffalo	2012	92.99% (KR858301)	93.96% (EU726442)	90.74% (EU726385)	96.29% (M81594)	93.02% (M14763)
6	STENT3	Buffalo	2012	93.95% (KR858301)	95.88% (EU726442)	92.18% (EU726385)	96.29% (M81594)	93.02% (M14763)
7	SPT-CB1	Buffalo	2013	97.91% (JF894242)	98.33% (FM162580.)	94.56% (EU726386)	94.97% (M81594)	92.97% (M14763)
8	STENT2	Buffalo	2012	93.96% (KR858301)	93.98% (EU726442)	90.37% (EU726386)	94.07% (M81594)	90.33% (M14763)
9	STENT4	Buffalo	2012	96.57% (KR858299)	97.01% (FM162581)	93.16% (EU726386)	94.78% (M81594)	91.52% (M14763)
10	SBWNT	Buffalo	1998	93.90% (KR858299)	95.31% (FM162581)	92.43% (EU726386)	99.18% (M81594)	99.09% (EU155058)
11	TBN-EJ	Cattle	2003	92.95% (KR858299)	94.74% (L07805)	91.46% (EU726386)	95.74% (M81594)	91.76% (M14763)
12	PDE	Cattle	1986	95.40% (KR858299)	95.83% (FM162581)	92.92% (EU726386)	98.45% (M81594)	98.39% (EU155058)
13	ASH	Buffalo	1992	97.06% (KR858299)	97.49% (FM162581)	93.72% (EU726386)	95.21% (M81594)	91.23% (M14763)
14	SB-PR	Buffalo	2012	97.12% (KR858301)	96.63% (EU726436)	93.27% (EU726385)	94.07% (M81594)	91.07% (M14763)
15	SB-RS	Buffalo	2014	97.56% (KR858299)	98.05% (FM162581)	93.66% (AF068701)	96.98% (AY918061)	96.97% (M14763)
16	SB-RHL	Buffalo	2014	97.14% (KR858299)	98.10% (EU726444)	94.29% (EU726386)	nd	nd
17	SB-RD	Buffalo	2014	97.51% (KR858301)	98.01% (EU726442)	93.53% (EU726385)	96.06% (M81594)	94.78% (M14763)
18	SB-RM	Buffalo	2014	97.12% (KR858301)	97.12% (EU726442)	93.27% (EU726385)	95.51% (M57459)	93.84% (M14763)
19	ERK-SC2	Bx Cattle	1986	97.73% (KR858301)	97.73% (FM162578)	94.09% (EU726385)	96.62% (M81594)	95.75% (M14763)
20	BTN06	Buffalo	2014	91% (OU830658)	91.47% (MF093650)	91.47% (EU726393)	97.20% (M57462)	96.25% (M14763)
21	BTN07	Buffalo	2014	95% (JF894242)	95% (KC257412)	90.50% (EU726385)	96.90% (M57462)	96.26% (M14763)
22	BTN08	Buffalo	2014	96.50% (KR858301)	96.50% (FM162578)	93.50% (EU726385)	94.88% (M57462)	93.64% (M14763)
23	BTN09	Buffalo	2014	96.14% (KR858301)	97.10% (FM162580)	94.20% (EU726386.)	94.72% (M57462)	93.44% (M14763)
24	BTN10	Buffalo	2014	93.81% (JF894242)	93.81% (KC257412)	90.45% (EU726386)	95.87% (M57462)	94.89% (EU155058)
25	BTN14	Buffalo	2014	96% (KR858299)	97% (FM162581)	93.50% (EU726386)	96.63% (M81594)	96.63% (EU155058)
26	BTN15	Buffalo	2014	95.50% (OU830658)	96% (EU726434)	93.50% (EU726386)	95.80% (M81594)	95.80% (EU155058)
27	BTN16	Buffalo	2014	94.76% (JF894242)	95.24% (L07805.)	93.33% (EU726386)	96.89% (M57462)	95.62% (M14763)
28	BRBS-1	Buffalo	2017	nd	nd	nd	98.79% (M81594)	98.32% (EU155058)
29	BRBS-2	Buffalo	2017	95.05% (AB551915)	96.04% (EU726442)	90.59% (EU726393)	95.11% (M81594)	95.11% (EU155058)
30	BRBS-3	Buffalo	2017	89.80% (JF894242)	91.33% (OM932504)	84.69% (EU726385)	93.67% (M57462)	92.42% (M14763)
31	BRBS-4	Buffalo	2017	92.79% (OU830658)	93.75% (KC257412)	91.83% (EU726386)	95.54% (M57462)	94.49% (EU155058)
32	BRBS-5	Buffalo	2017	95% (KC257411)	95% (KR858301)	91.50% (EU726385)	95.34% (M57462)	94.38% (EU155058)
33	BRBS-6	Buffalo	2017	94.21% (JF894242)	95.79% (FM162580)	92.63% (EU726386)	94.79% (M57462)	93.74% (EU155058)
34	BRBS-7	Buffalo	2017	94.36% (KR858301)	94.36% (EU726442)	89.74% (EU726393)	94.25% (M81594)	93.61% (M14763)
35	BRBS-8	Buffalo	2017	95% (KR858301)	96% (EU726442)	91% (EU726393)	94.69% (M81594)	94.69% (EU155058)
36	BRBS-9	Buffalo	2017	98.11% (OU830658)	98.11% (EU726440)	97.17% (EU726385)	95.17% (M81594)	94.56% (M14763)
37	BRBS-10	Buffalo	1996	92.38% (JF894242)	92.86% (FM162576)	90% (EU726386)	97.80% (M57462)	96.52% (M14763)
36	BRBS-13	Buffalo	1996	95% (JF894242)	95.50% (EU726435)	94% (EU726386)	95.03% (M57462)	93.75% (M14763)
39	BYW-EJ	Cattle	1992	95.63% (KR858301)	96.60% (EU726442)	91.75% (EU726393)	98.17% (M81594	97.56% (M14763)
40	BKN-EJ2	Buffalo	1988	96.47% (AB551914)	96.82% (FM162578)	94.35% (EU726385)	97.82% (M81594	97.82% (EU155058)

**Notes:** nd = not done.

φ accession number is the highest query coverage and sequence similarity only;

* isolate number 1-14 generated from Subekti et al. (2023).

In the second step, the MINI primers are used to further refine the species identification by eliminating one of the three possible species in the *Trypanozoon* subgenus. The MINI primer pair has been reported to amplify the *gRNA-kDNA* minicircle gene in *T. evansi* but not *T. brucei* (Artama et al., 1992). The nucleotide sequences of these PCR amplicons showed sequence similarities to *T. evansi*, ranging from 93.67% to 99.18%, and *T. equiperdum*, ranging from 90.33% to 99.09%; none showed sequence similarity to *T. brucei* (Table 2). This result provides additional information not mentioned by Artama et al. (1992), whose study did not include *T. equiperdum*. At the same time, PCR using the RoTat 1.2-a primer pair (Table 3) showed positive results for all isolates. These results are consistent with Claes et al. (2004), who explained that the RoTat 1.2 primer pair (RoTat 1.2-a in this study) amplified 100% (8/8) of *T. evansi* and 77.8% (7/9) *T. equiperdum* isolates but no *T. brucei* isolates. It can be concluded that the 43 trypanosome isolates were likely *T. evansi* or *T. equiperdum* and not *T. brucei*.

**Tabel 3:** The results of the PCR test for Indonesian trypanosome isolates used different primers for identification and genotyping.

No	Code	District, Province	Host	ESAG 6/7*	MINI^*^	MAXI	EVAB*	RoTat 1.2-a*	RoTat 1.2-b	RoTat 1.2-c	Genotype
1	AMN-SB1	Amuntai, South Kalimantan	Buffalo	+	+	-	-	+	-	+	non A/B
2	KPG	Kulon Progo, Yogyakarta	Buffalo	+	+	-	-	+	+	+	A
3	PML	Pemalang, Central Java	Buffalo	+	+	-	-	+	-	+	non A/B
4	STENT1	East Sumba, East Nusa tengara	Buffalo	+	+	-	-	+	+	-	A
5	STENT5	East Sumba, East Nusa tengara	Buffalo	+	+	-	-	+	+	+	A
6	STENT3	East Sumba, East Nusa tengara	Buffalo	+	+	-	-	+	-	+	non A/B
7	SPT-CB1	Sampit, Central Kalimantan	Buffalo	+	+	-	-	+	+	+	A
8	STENT2	East Sumba, East Nusa tengara	Buffalo	+	+	-	-	+	+	+	A
9	STENT4	East Sumba, East Nusa tengara	Buffalo	+	+	-	-	+	+	+	A
10	SBWNT	Sumbawa, West Nusa Tenggara	Buffalo	+	+	-	-	+	+	+	A
11	TBN-EJ	Tuban, East Java	Cattle	+	+	-	-	+	+	+	A
12	PDE	Pidie, Aceh	Cattle	+	+	-	-	+	-	+	non A/B
13	ASH	Asahan, North Sumatra	Buffalo	+	+	-	-	+	+	+	A
14	SB-PR	East Sumba, East Nusa tengara	Buffalo	+	+	-	-	+	+	+	A
15	SB-RS	East Sumba, East Nusa tengara	Buffalo	+	+	-	-	+	+	+	A
16	SB-RHL	East Sumba, East Nusa tengara	Buffalo	+	+	-	-	+	+	+	A
17	SB-RD	East Sumba, East Nusa tengara	Buffalo	+	+	-	-	+	+	+	A
18	SB-RM	East Sumba, East Nusa tengara	Buffalo	+	+	-	-	+	+	+	A
19	ERK-SC2	Enrekang, South Sulawesi	Bx Cattle	+	+	**-**	-	+	+	+	A
20	BTN06	Pandeglang, Banten	Buffalo	+	+	**-**	-	+	+	+	A
21	BTN07	Pandeglang, Banten	Buffalo	+	+	**-**	-	+	+	+	A
22	BTN08	Pandeglang, Banten	Buffalo	+	+	**-**	-	+	+	+	A
23	BTN09	Pandeglang, Banten	Buffalo	+	+	**-**	-	+	+	+	A
24	BTN10	Pandeglang, Banten	Buffalo	+	+	**-**	-	+	+	+	A
25	BTN13	Pandeglang, Banten	Buffalo	+	+	**-**	-	+	+	+	A
26	BTN14	Pandeglang, Banten	Buffalo	+	+	**-**	-	+	+	+	A
27	BTN15	Pandeglang, Banten	Buffalo	+	+	**-**	-	+	+	+	A
28	BTN16	Pandeglang, Banten	Buffalo	+	+	**-**	-	+	+	+	A
29	BRBS-1	Brebes, Central Java	Buffalo	+	+	**-**	-	+	+	+	A
30	BRBS-2	Brebes, Central Java	Buffalo	+	+	**-**	-	+	+	+	A
31	BRBS-3	Brebes, Central Java	Buffalo	+	+	**-**	-	+	+	+	A
32	BRBS-4	Brebes, Central Java	Buffalo	+	+	**-**	-	+	+	+	A
33	BRBS-5	Brebes, Central Java	Buffalo	+	+	**-**	-	+	+	+	A
34	BRBS-6	Brebes, Central Java	Buffalo	+	+	**-**	-	+	+	+	A
35	BRBS-7	Brebes, Central Java	Buffalo	+	+	**-**	-	+	+	+	A
36	BRBS-8	Brebes, Central Java	Buffalo	+	+	**-**	-	+	+	+	A
37	BRBS-9	Brebes, Central Java	Buffalo	+	+	**-**	-	+	+	+	A
38	BRBS-10	Brebes, Central Java	Buffalo	+	+	**-**	-	+	+	+	A
39	BRBS-13	Brebes, Central Java	Buffalo	+	+	**-**	-	+	+	+	A
40	SBWNT2	Sumbawa, West Nusa tenggara	Cattle	+	+	**-**	-	+	+	+	A
41	BYW-EJ	Banyuwangi, East Java	Bx Cattle	+	+	**-**	-	+	+	+	A
42	BKN-EJ2	Bangkalan, East Java	Buffalo	+	+	**-**	-	+	+	+	A
43	BYW-EJ2	Banyuwangi, East Java	Cattle	+	+	**-**	-	+	+	+	A

**Notes:** Bx Cattle = Brahman Cross Cattle.

* isolate number 1-14 generated from Subekti et al. (2023). + = PCR positive; – = PCR negative;

MINI = gRNA-kDNA minicircle; MAXI = ND5-kDNA maxicircle; RoTat 1.2-b = ILO7957/8091; RoTat 1.2-c = TeRoTat920/1070 (see Table 1).

The third and final species identification step used the MAXI primer pair to PCR amplify the kDNA maxicircle gene. The MAXI primer pair has been reported to amplify only the kDNA maxicircle genes in *T. equiperdum* and *T. brucei* (Li et al., 2007; Suganuma et al., 2016). Since *T. evansi* has lost the maxicircle gene, it cannot be amplified by the MAXI primer pair. PCR using the MAXI primer pair was negative for all isolates, supporting the identification of *T. evansi* and excluding *T. equiperdum* (Figure 1 and Table 3).

The fourth step is an additional step to determine the *T. evansi* genotype, which will be identified as type A with a positive result with the ILO7957/8091 primer pair (RoTat 1.2-b in this study) and negative a result with the EVAB primer pair, while *T. evansi* type B shows the opposite results (Njiru et al., 2006; Birhanu et al., 2016; Boushaki et al., 2019). *T. evansi* types A and B are differentiated based on minicircle kDNA (Cuypers et al., 2017). The immunodominant RoTat 1.2 variable surface glycoprotein is primarily used to identify *T. evansi* type A, while EVAB primer is primarily used to identify *T. evansi* type B isolates based on present or absent of B minicircle kDNA (Birhanu et al., 2016; Cuypers et al., 2017; Boushaki et al., 2019). To date, *T. evansi* type B has only been reported in Eastern Africa, probably present but not detected in Western and Northern Africa (Cuypers et al., 2017). However, there have been reports that *T. evansi* type B has only been isolated from camels and found in a limited geographic area, especially Kenya, Ethiopia (both are Eastern Africa), and Sudan which is known to belong to parts of Northern Africa (Njiru et al., 2006; Njiru et al., 2011; Birhanu et al., 2016). In contrast, *T. evansi* type A has been frequently isolated from various hosts in Africa, South America, and Asia (Birhanu et al., 2016; Behour & Abd El Fattah, 2023).

Previous research has shown that the KETRI 2472 isolate was misclassified, and it has been suggested that it should be reviewed. The KETRI 2472 isolate originates from camels in Sudan and is currently believed to be *T. evansi* type A (Njiru et al., 2006). However, since data from Njiru et al. (2006) showed that this isolate was negative for RoTat 1.2 and EVAB, so it probably deserved to be classified as *T. evansi* type non-A/B. *T. evansi* non-A/B (KETRI 3552 and 3557) has also been reported in Kamidi et al. (2017) but there have been several criticisms of the identification approach. The KETRI 3552 and 3557 were both classified as *T. evansi* non-A/B despite being PCR positive for RoTat 1.2. There are some criticisms, first, they do not prove whether PCR is positive or not for B minicircles which is the key to identifying *T. evansi* type B. Second, only relied on A-281-del as a genetic marker and did not consider RoTat 1.2 (using ILO7957/ILO8091 primer set) as the key to identifying *T. evansi* type A, lead doubts and confusion regarding identification and assignment the true status of KETRI 3552 and 3557. Carnes et al. (2015) reported that *T. evansi* with negative RoTat 1.2 is likely type B, C or something else. This evidence shows that A-281-del as the main key identification for type A is not appropriate, so KETRI 3552 and 3557 should be categorized as *T. evansi* type A. Third, Kamidi et al. (2017) doubted the RoTat 1.2 primer (ILO7957/ILO8091) because it could not detect all *T. evansi*. This is actually supporting evidence that RoTat 1.2 (ILO7957/ILO8091) is able to differentiate *T. evansi* type A and others (type B or something else). This also happened in our study where four out of 43 isolates showed negative with the primers ILO7957/ILO8091 (RoTat 1.2b in this study). This finding is similar with Ngaira et al. (2004) which only detected positive 72.22% of *T. evansi* tested using same primer sets. In contrast, the use of another RoTat 1.2 primer (RoTat 1.2a in this study) proved successful in detecting all *T. evansi* that had been tested as reported by Claes et al. (2004) and in this study.

This study identified four out of 43 (9.30%) *T. evansi* isolates as negative with both the EVAB and ILO7957/8091 primer pairs and could possibly be considered for classification as non-A and non-B types (non-A/B; Table 3). The *T. evansi* type non-A/B isolates (PML, AMN-SB1, and STENT3) were isolated from buffalo, while the PDE isolate was isolated from cattle. This is the first study to isolate *T. evansi* type non-A/B strains from bovines outside of Africa. The STENT1 isolate was classified as *T. evansi* type A because it showed a positive ILO7957/8091 result. Based on the report by Behour & Abd El Fattah (2023), which classified *T. evansi* type B based on a negative TeRoTat920/1070 result (RoTat 1.2-c in this study) and a positive EVAB result, STENT1 may also be considered *T. evansi* type non-A/B because TeRoTat920/1070 and EVAB are both negative. However, we consider the classification of STENT1 as non-A/B type to be inappropriate because the sensitivity of TeRoTat920/1070 is below that of ILO7957/8091. Salim et al. (2011) reported that the TeRoTat920/1070 primer pair could amplify the VSG RoTat 1.2 gene belonging to *T. evansi* in 63.3% (19/30) of isolates, while 36.7% (11/30) were negative. Overall, the difference in detection of three RoTat 1.2 primer sets from this study and other studies seems to require a more in-depth study regarding the identification of *T. evansi* type A.

**Figure 2:**
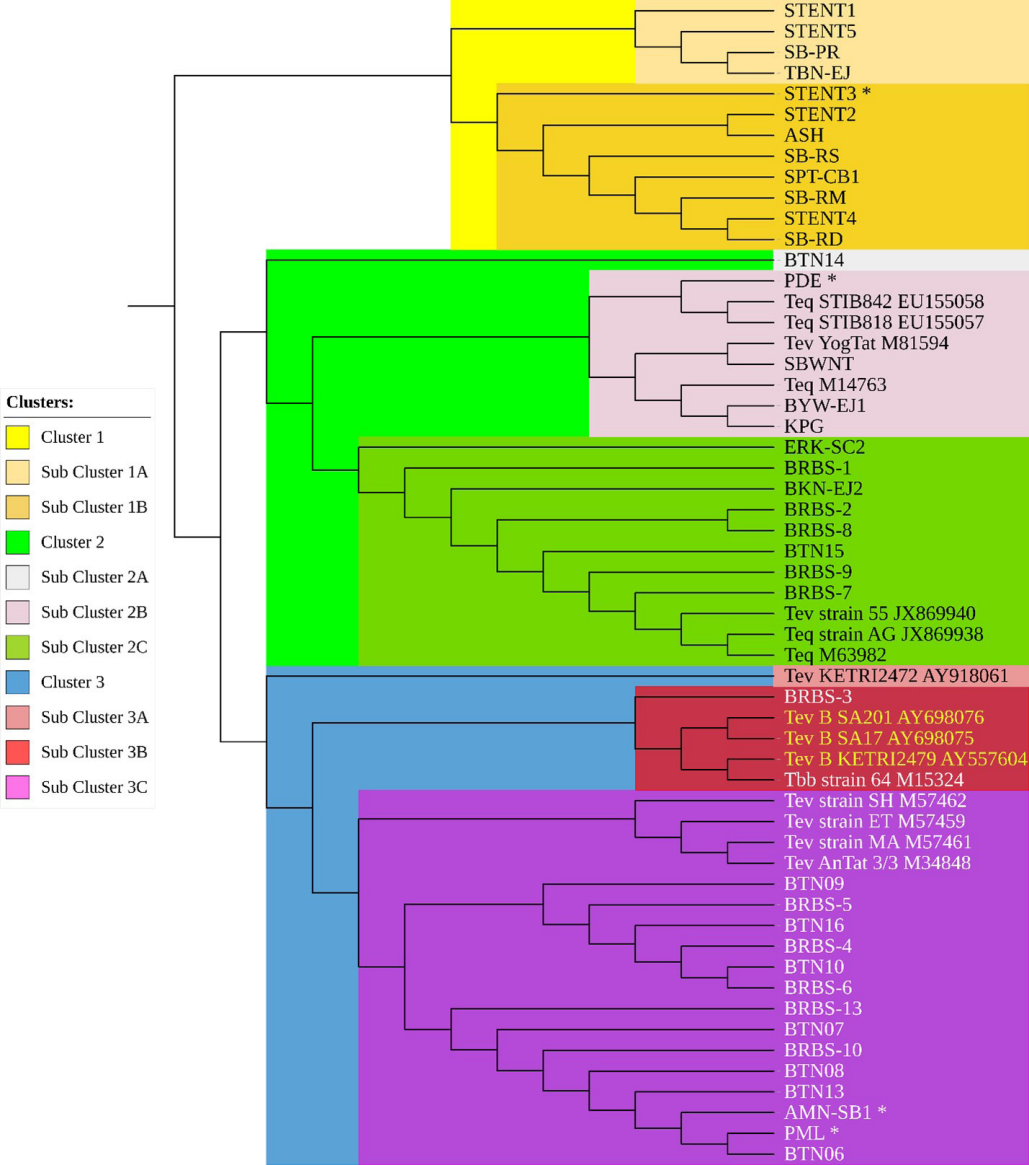
*Trypanozoon* cladogram based on the nucleotide sequence of the *gRNA-kDNA* minicircle gene constructed with Neighbor-joining method using Jukes-Cantor nucleotide distance measurement and bootstrap analysis with 1000 replicates. The asterisk is *T. evansi* type non-A/B. Tev = *Trypanosoma evansi*, Tev B = *Trypanosoma evansi* type B, Tbr = *Trypanosoma brucei*, and Teq = *Trypanosoma equiperdum*.

**Figure 3:**
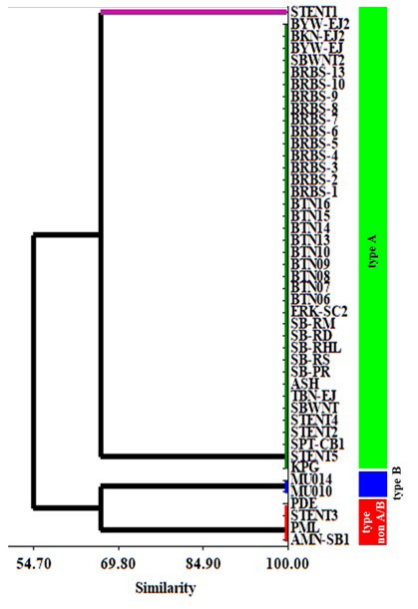
*Trypanosoma evansi* cladogram constructed using the average linkage method with squared Euclidean distance measurement. Binary data conversion of *T. evansi* type B isolates (MU014 and MU010) generated from Birhanu et al. (2016).

A cladogram constructed based on the nucleotide sequence of the *gRNA-kDNA* minicircle shows that two *T. evansi* type non-A/B isolates from Indonesia (PML and AMN-SB1) are grouped into Cluster 3 with other *T. evansi* type A and B isolates and the KETRI 2472 isolate (Figure 2). The other Indonesian *T. evansi* type non-A/B (STENT3 and PDE) were grouped into Cluster 1 and 2 respectively (Figure 2). This approach was unsuccessful in classifying each *T. evansi* genotype separately. A suggested alternative approach for cladogram construction was to use HCA based on binary data derived from positive or negative observational data obtained from nucleic acid amplification using the primer pairs ESAG6/7, MINI, TeRoTat920/1070, ILO7957/8091, and EVAB. The cladogram constructed using HCA successfully grouped *T. evansi* type A, B, and non-A/B isolates into separate clusters (Figure 3). However, one weakness of this approach is that it cannot explore and classify genetic diversity in more detail based on the nucleotide or amino acid sequences of each isolate.

### Phylogeography of Indonesian *T. evansi*

**Figure 4:**
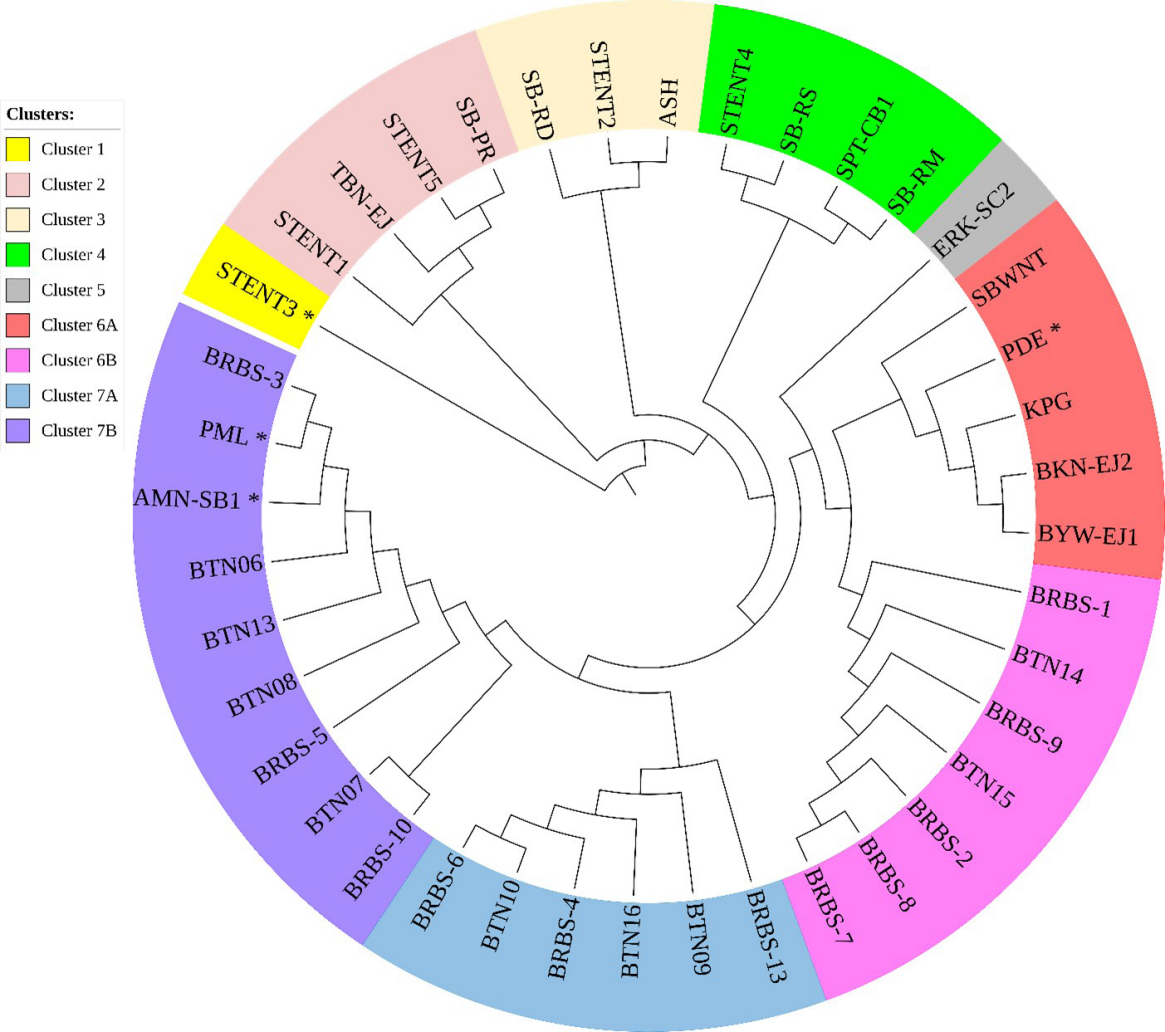
Cladogram of Indonesian *Trypanosoma evansi* based on the nucleotide sequence of the *gRNA-kDNA* minicircle gene constructed using Neighbor-joining method using Jukes-Cantor nucleotide distance measurement and bootstrap analysis with 1000 replicates. The asterisk indicates *T. evansi* type non-A/B in this study

*T. evansi* isolates from Indonesia were generally grouped into seven clusters based on the nucleotide sequence of the *gRNA-kDNA* minicircle (Figure 4). Cluster 6 was divided into two sub-clusters containing isolates from six provinces i.e. Aceh (PDE), Banten (BTN), Central Java (BRBS), Yogyakarta (KPG), East Java (BKN-EJ2, BYW-EJ1), and West Nusa Tenggara (SBWNT). Cluster 7 was divided into two sub-clusters, with isolates originated from three provinces i.e Banten (BTN), Central Java (BRBS, PML), and South Kalimantan (AMN-SB1). Meanwhile isolates from East Nusa Tenggara province (STENT, SB-PR, SB-RD, SB-RM, SB-RS) were grouped into clusters 1 to 4 together with isolates from North Sumatra province (ASH, cluster 3), East Java province (TBN-EJ, cluster 2) and Central Kalimantan province (SPT-CB1, cluster 4).

**Figure 5:**
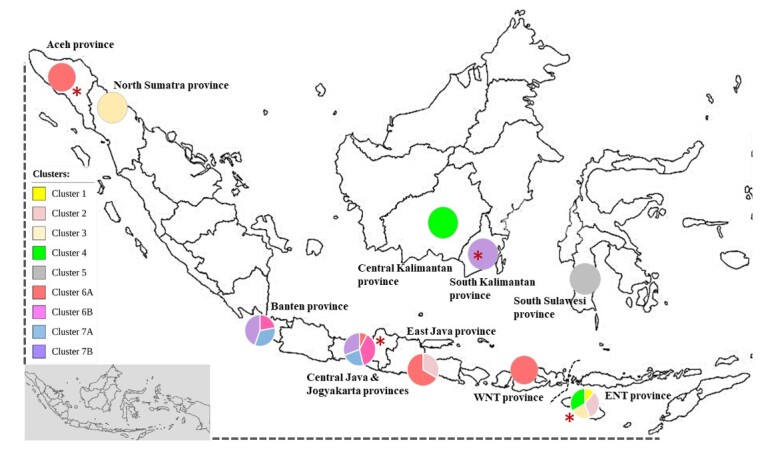
Distribution map of the *Trypanosoma evansi* sub-cluster in Indonesia based on the genetic diversity of the minicircle gene (Figure 4). The red asterisk indicates the origin of *T. evansi* type non-A/B in this study. WNT province = West Nusa Tenggara province, ENT province = East Nusa Tenggara province.

Geographically, the four *T. evansi* type non-A/B isolates originate from a province that historically did not have trade routes related to livestock movement (Figure 5), especially buffalo and cattle. The four *T. evansi* types non-A/B isolates were also isolated over a long period. Therefore, the most likely hypothesis was that they emerged independently in each region. While the *T. evansi* type non-A/B isolates in South Kalimantan and East Nusa Tenggara (ENT) provinces were isolated in adjacent years, these provinces do not have historical and current buffalo trade routes.

The areas with high genetic diversity are the provinces of East Nusa Tenggara, Central Java (including Yogyakarta), and Banten (Figure 5). Central Java (including Yogyakarta) provinces had four sub-clusters (6A, 6B, 7A, and 7B), while Banten province had three sub-clusters (6B, 7A, and 7B). Historically, livestock movement between Banten and Central Java provinces (vice versa), especially cattle and buffalo, has existed for a long time, it is possible that the isolates from the two provinces originated from same ancestor.

The *T. evansi* isolates isolated from buffaloes in the ENT province showed interesting patterns. They were all grouped into the same cluster (Cluster 1, Figure 2) when compared with *T. evansi* isolates from outside Indonesia or separated into four cluster when compared with isolates from Indonesia (Figure 4). All isolates from the ENT province were isolated in 2012 from buffaloes that survived the Surra outbreak in 2010–2012. The Surra outbreak in ENT Province in 2010–2012 killed more than 1700 horses and buffaloes (Subekti & Yuniarto, 2020). Based on their clustering (Figure 5), isolates from ENT appear to be related to isolates from East Java, Central Kalimantan and North Sumatra provinces. However, the difference in the year of origin of ENT isolates and isolates from East Java and North Sumatra provinces is greatly different, 2013 versus 1992. Unfortunately, data on the historical spread of trypanosomes at that time are unavailable, making it difficult to predict the association among isolates from those provinces. The genetic relationship between ENT isolates and isolates from Central Kalimantan and East Java provinces also cannot be confirmed conclusively, even though historically livestock movement from ENT to these two provinces has existed for a long time. Further studies are needed to reveal the distribution of *T. evansi* between islands by comparing data on animal movements between them in the same or adjacent years.

## Conclusions

Forty-three trypanosoma isolates from Indonesia were identified as *Trypanosoma evansi* using a molecular identification algorithm. Further identification showed that 39 isolates were type A and 4 isolates were possibly non-A/B types. This study reports the first isolation of *T. evansi* which is suspected to be type non-A/B from bovines. Non-A/B type of *T. evansi* was found in isolates originating from the provinces of Aceh, Central Java, South Kalimantan and East Nusa Tenggara. This study is also the first to report high genetic diversity in the Banten, Central Java, and East Nusa Tenggara provinces based on the nucleotide sequences of the *gRNA-kDNA* minicircle. Further research is needed to uncover and more deeply exploration.
